# Function or outcomes based performance for public health systems?

**DOI:** 10.1186/2045-4015-1-47

**Published:** 2012-11-26

**Authors:** Fiona Sim

**Affiliations:** 1 Department of Health Services Research & Policy, London School of Hygiene and Tropical Medicine, 15-17 Tavistock Place, London, WC1H 9SH, UK

## Abstract

This commentary considers the merits of exploring different public health delivery systems among developed countries to consider which models are most effective. It challenges the conventional focus on delivery of services or functions and asks why we are not primarily interested in delivery of better public health outcomes for our populations. Achieving these outcomes requires the commitment of all sectors of our respective communities and the deployment of a range of delivery systems tailored to the national political and cultural context.

## Introduction

The paper by Scutchfield et al. [1] presents very interesting reading about parallel initiatives in the US and Israel to make public health services more efficient and effective. Whilst we can learn from the US-Israel comparison, the authors are right to suggest that further international collaboration could be fruitful in understanding what makes an optimally effective public health system. However, the likelihood is that one size will not fit all jurisdictions.

What is perhaps not surprising, but opens the way to further collaboration (not only between these two countries, but further afield) is the paucity of evidence in the developed world about what influences actual health outcomes. Ultimately, the focus should not be on proxy measures expressed as processes and outputs, but rather on real changes to health status resulting from public health interventions. So whilst a consideration of measurable targets is desirable, should those targets be process driven, as is commonly the case, or should they be real changes in health status, which might be achieved by a variety of different processes to fit the local or regional context?

### Learning from history

It is fascinating to read about the early public health services in US ports and their aims; go a little earlier – back to 1753 in fact [2] - and you will discover that it was on ships leaving ports that some very important public health discoveries were made. Notable among them was the discovery that a supply of citrus fruit could prevent scurvy amongst sailors. This discovery was made by James Lind, a ship’s surgeon, as a result of what would now probably be described as observational epidemiology. Notable, too, is the observation by Scutchfield et al., that the public health movement was progressed by people such as Shattuck in 1850, a bookseller without formal public health expertise, but with a passion to improve the health of the population and in particular of the poorest. The parallel in the UK was perhaps Edwin Chadwick, who led the introduction of transformational social reforms in 1840s England, heralding the Public Health Act of 1848 that introduced statutory public health services [3].

Services similar to the USA’s ten essential public health services are seen elsewhere in the developed world, with each nation declaring its own priorities and thus defining their essentials differently.

As long as there is no clear internationally accepted definition of a public health service this variation is likely to continue. Interestingly, definitions of public health services rarely refer to the very broad WHO definition of health –“Health is a state of complete physical, mental and social well-being and not merely the absence of disease or infirmity.” [4]. In contrast, Winslow’s widely accepted definition of public health, "the science and art of preventing disease, prolonging life and promoting health through the organized efforts and informed choices of society, organizations, public and private, communities and individuals" [5] continues to be highly relevant to modern societies. It presents a vision requiring ownership and commitment by all sectors of society and goes far beyond the provision of the programmed services usually recognised as ‘public health’ services.

Israel's current public health system developed from the service delivered under British mandate in the period prior to independence. Whilst in the UK the public health system has been reconfigured many times since 1948, usually as a result of political and fiscal priorities, it seems that in Israel it has been encouraged to develop and flourish as an integral element towards ensuring the country’s wellbeing. In the USA, the focus has been on delivery of public health services, from the three described in the 1988 IOM report on the Future of Public Health to the ten Essential Public Health Services, first described by CDC in 1994.

### Evolution of public health delivery

The next stage in the evolution of public health services should, surely, be the delivery of evidence-based services that will achieve better health for the population they serve. Whilst this may seem obvious, the requirement to maintain ten [or any other number] essential functions or services may, unintentionally, present constraint to innovation in the achievement of improved health. Indeed, as pointed out by Marmot [6] we may elicit negative consequences through ill-planned delivery. Health inequalities widen rather than narrow, if the poorest are able to benefit less than others.

So, while a service-based approach may be pragmatic, an outcomes based focus must surely be the ultimate aim of public health systems. In England the recent publication of the first Public Health Outcomes Framework [7] is a step towards this goal. It is perhaps less than perfect and several measures are yet to be developed. Nonetheless, it does recognise the critical role of functions and services extending way beyond the conventional public health system that must be harnessed if we are to see better health and reduced inequalities for the population. So for example, there are expectations that the education, justice, social care, environment and transport sectors all play their parts in delivering improved public health.

The domains of the Public Health Outcomes Framework (England) are:

1. Improving the wider determinants of health

2. Health improvement

3. Health protection

4. Healthcare Public Health and prevention of premature mortality

It is of note that the Framework makes no reference to *how* outcomes are to be achieved; that is for national and local discretion. However, it does set out *what* is to be achieved. This may well result in some significant variation in delivery systems, but if we agree on the principle of achieving intended outcomes, the exact mechanism of reaching those outcomes is less critical. This means that ten functions or ten services are less important than the achievement of improved health. Globally, this must be linked with reduction of health inequalities.

### Opportunities for international collaboration

Given very different national priorities, the governmental contribution to public health will vary substantially across countries. One valuable tool is the ‘Ladder of Intervention’ developed by the Nuffield Council on Bioethics, which sets out the range of possible government intervention in public health, from statutory regulation at one end to complete freedom of personal choice (no government action) at the other [8]. At any time, countries vary widely in the level of government intervention in place to protect and promote public health. Indeed, it has been argued recently that modern democracies may be pursuing agendas that are not compatible with improving the health of their citizens [9].

Clearly, nowhere are we starting with a blank canvas on which to paint our new public health system and so it has to evolve over time. In Israel the link with HMOs, and thus with health care delivery, is described eloquently by Scutchfield et al. Similarly in the UK, family doctors’ contracts with the National Health Service include elements of public health delivery, particularly in relation to the management of patients with long term conditions, the administration of immunisations and other preventive measures. In the US, the public health system has been historically less connected with health care and the delivery of better population health viewed as a separate entity. Recent initiatives (in the US, China and elsewhere) to make connections between health care and public health are beginning to break down such artificial barriers [10].

One risk of emphasising the fact that public health is everybody’s business is that it may become nobody’s priority. So, however the public health system is delivered, making it a priority is essential. One might think that the macroeconomic value of population health should be sufficient to ensure public health's priority status, but history has demonstrated that this is not necessarily the case. However public health services are delivered, we must not allow responsibility for public health to be attributed solely to designated programmatic public health services or functions.

So, Scutchfield et al. are correct in suggesting that there is potential benefit for other countries interested in joining efforts to clarify the role, scope, and functions of public health. As they suggest, carefully selected process measures should play an important role in achieving better health outcomes, and international collaboration to identify the most effective processes, via comparative evaluation, is to be welcomed. We know from a recent article in this journal that in healthcare, international comparisons can be made effectively to target interventions to reduce mortality where identified as amenable to health care [11]: so why not a similar project for public health? The following caveat must, however, be kept in mind: these efforts must acknowledge that meaningful public health outcomes will be achieved only by recognising the need for an inclusive approach to the scope of public health that extends well beyond conventional public health services.

## 

**Commentary** on Scutchfield FD, Miron E, Ingram R. From Service Provision to Function Based Performance – Perspectives on Public Health Systems from the USA and Israel. Israel Journal of Health Policy Research.

## Competing interests

The author declares that she has no competing interests.

## Author information

Fiona Sim is a public health physician and family doctor in the UK. She is currently: Chair, Royal Society for Public Health; Visiting professor, University of Bedfordshire; Clinical research fellow, London School of Hygiene & Tropical Medicine; Cluster Medical Director, NHS Bedfordshire & Luton; part-time general medical practitioner, Bedfordshire, UK; Joint Editor-in-Chief, ***Public Health***. Previously, she was Head of Public Health Development, Department of Health, England.
